# Comparison of patient-reported outcomes measurement information system and legacy instruments in multiple domains among older veterans with chronic back pain

**DOI:** 10.1186/s12891-020-03587-6

**Published:** 2020-09-08

**Authors:** Rabih Nayfe, Matthieu Chansard, Linda S. Hynan, Eric M. Mortensen, Thiru Annaswamy, Liana Fraenkel, Una E. Makris

**Affiliations:** 1 Department of Internal Medicine, UT Southwestern (UTSW) Medical Center, Dallas, TX USA; 2 Department of Population and Data Sciences, UTSW, Dallas, TX USA; 3 Department of Psychiatry, UTSW, Dallas, TX USA; 4 Department of Medicine, University of Connecticut, Farmington, CT USA; 5 Department of Medicine, VA North Texas Health Care System, Dallas, TX USA; 6 Department of Physical Medicine & Rehabilitation, VA North Texas Health Care System, Dallas, TX USA; 7 Department of Medicine, VA Connecticut Healthcare System, West Haven, CT USA; 8 Department of Medicine, Yale University School of Medicine, New Haven, CT USA; 9 Department of Internal Medicine, Division of Rheumatic Diseases, VA North Texas Health Care System, 4500 S Lancaster Rd., Dallas, TX 75216 USA

**Keywords:** Older adults, Chronic back pain, Patient reported outcomes, Construct validity, Measurement

## Abstract

**Background:**

Chronic low back pain (cLBP) results in significant physical, psycho-social and socioeconomic burden*.* Identifying efficient and reliable patient reported outcome measures is critical for research and clinical purposes. The NIH’s Patient Reported Outcomes Measurement Information System (PROMIS) instruments have not been compared to validated “legacy” instruments in older adults with cLBP. This study evaluates construct (convergent and discriminant) validity and time to complete (TTC) PROMIS as compared to legacy instruments.

**Methods:**

We enrolled older Veterans (age 60+) with cLBP with/without leg pain scheduled for lumbar epidural steroid injections. Subjects completed PROMIS computer adaptive test item banks and corresponding legacy instruments in the following domains: pain intensity, interference, and behavior; functional status; depression and anxiety; fatigue; sleep and social functioning. Convergent and discriminant validity between PROMIS and legacy instruments was evaluated using Spearman rank order correlations; Mann-Whitney U tests compared TTC.

**Results:**

Of the 71 Veterans recruited, the median (IQR) age was 67 (63–71) years old, 94% were men, 76% were White, 17% Black, and 96% were Non-Hispanic. Spearman correlations between PROMIS and legacy instruments showed moderate to very strong convergent validity in all domains (*r* = 0.4–1.0), except for social functioning and pain behavior (PROMIS Pain Behavior with Fear Avoidance Belief Questionnaire). The total median TTC for all PROMIS items was significantly shorter than legacy items, 8 min 50 s vs 29 min 14 s respectively, *p* < 0.001.

**Conclusions:**

Given time efficiency of using PROMIS, along with strong construct validity, PROMIS instruments are a practical choice for measuring multidimensional PROs in older Veterans with cLBP for both research and clinical purposes.

## Background

Back pain in older adults (>65 years of age) is an important public health problem with significant physical and psycho-social consequences. According to the Global Burden of Disease Study 2017, low back pain is the single leading cause of disability worldwide since 1990 with an increase in years lived with disability by 17.5% since 2007 [1], with lifetime prevalence exceeding 80%, and chronic back pain developing in 23% [2]. Moreover, back pain incurs high socioeconomic burden, with total health expenditures in the US exceeding $100 billion for direct and indirect costs related to back pain [3]. Costs related to chronic back pain are expected to rise as the population with chronic back pain ages [4, 5].

In general, chronic pain has been linked to limitation in mobility and daily activities, opioid dependency, anxiety and depression, and decreased quality of life [6]. Therefore, in order to most effectively assess and manage back pain in older adults, it is important to understand the biopsychosocial context and consequences in this population [7–9]. Chronic low back pain (cLBP) in older adults is complex: there are multiple contributing factors to pain and pain-associated disability including anxiety, depression, cognitive impairment and psychological maladaptation (fear avoidance beliefs and catastrophizing) [7, 10, 11]. Leaders in the cLBP field have outlined a minimum set of outcomes that should be included in studies evaluating cLBP [9]. While various patient reported outcomes (PRO) measures have been validated and widely used throughout the back pain literature (hereafter referred to as “legacy” instruments), they are frequently criticized as being too burdensome to use in research and clinical practice [12]. Also, these instruments can be costly to obtain and time consuming to perform on a routine basis which led to the development of more accessible and efficient measures.

Since the early 2000’s, National Institutes of Health (NIH) has invested heavily to develop robust outcome measures, Patient Reported Outcomes Measurement Information System (PROMIS) to be applied across different medical conditions (http://www.healthmeasures.net/explore-measurement-systems/promis). The purpose of PROMIS is to provide clinicians and researchers with efficient, valid and reliable assessments of a patient’s health status derived from patient responses to a set of rigorously developed questions about different quality of life measures (physical, mental and social) [13–15]. PROMIS questions may be administered with computer adaptive testing (CAT), developed based on item response theory (IRT), where questions are dynamically administered based on a subject’s prior responses, thus maintaining precision while using the minimum number of questions [13, 16].

While PROMIS has been tested in various populations, including chronic pain (of which back pain was a subset), PROMIS instruments, in multiple domains, have not been compared to the validated legacy instruments in older adults or Veterans with cLBP. In this study we sought to assess the construct (convergent and discriminant) validity and time to complete (TTC) PROMIS as compared to legacy instruments in older Veterans with cLBP with or without leg pain. Our a priori hypothesis was that PROMIS would relate well with the corresponding legacy instruments and would be completed more efficiently.

## Methods

### Study design and population

This was a cross-sectional pilot study in which we recruited Veterans ≥60 years old with nonmalignant, noninfectious chronic (> 3 months duration) back pain with or without associated leg pain, who were referred and scheduled for an epidural steroid injection (ESI) from the Dallas VAMC Physical Medicine & Rehabilitation (PM&R) Spine Clinic (Table 1). Patients scheduled to receive the steroid injection were screened prior to the date of their procedure and if they met the inclusion criteria listed above, were introduced by the PM&R team and approached by the research staff to obtain informed consent and complete the surveys on the day of the ESI. Exclusion criteria were receiving an ESI within 3 months prior to this study, inability to speak English, severe cognitive impairment (failing a Mini-Cog test with total score < 3) [17] and back pain due to infectious or malignant etiology. This study was approved by the Dallas VA Institutional Review Board.

**Table 1:** Participant Demographics and Back Pain Characteristics

Patient characteristics: ***n*** = 71	n (%) or median [IQR]
Age, median [IQR]	67 [63–71]
Male, n (%)	67 (94.4%)
Race, n (%)	
White	54 (76%)
Black	12 (16.9%)
Other	3 (4.2%)
Declined	2 (2.8%)
Ethnicity, n (%)	
Non-Hispanic	68 (95.8%)
VA Service Connected Disability (MSK), n (%)	40 (56.3%)
Charlson Comorbidity Index, median [IQR]	4 [3–5]
BMI, median [IQR]	31.0 [27.1–35.0]
Depression, Anxiety and/or PTSD, n (%)	42 (59.2%)
Psychotropic Medication, n (%)	45 (63.4%)
**Back pain characteristics**	
Only Back Pain, n (%)	25 (35.2%)
Pain Intensity (Numerical Rating Scale), median [IQR]	5.5 [4–7]
Pain duration > 5+ years, n (%)	45 (63.4%)
Radicular symptoms, n (%)	66 (93%)
Analgesic Medication, n (%)	67 (94.4%)
Prior Epidural Steroid Injection > 3 months, n (%)	25 (35.2%)

### PROMIS and legacy measures

Participants completed the battery of tests (PROMIS CAT item banks and corresponding validated legacy instruments) (Table 2) prior to the ESI. Eligible participants were directed by research staff to an exam room with a dedicated desktop computer that was used to complete both sets of surveys. Research staff also documented field notes as participants completed the instruments (regarding usability or obstacles to completion). PROMIS instruments were administered as CATs using the PROMIS Assessment Center (www.assessmentcenter.net), and all of the legacy instruments were also administered on the computer for ease of use and ability to automatically measure time to completion, with exception of the graphics from Brief Pain Inventory (BPI), and the Numerical Rating Scale for Pain Intensity (NRS-PI). If the participant was not able to complete all sections of the PROMIS and legacy instruments, within each domain, prior to being called for their ESI procedure, these instruments were considered incomplete. We did not analyze responses from participants who had missing data for either PROMIS or legacy instruments. The presentation order of PROMIS and legacy instruments was randomized.

**Table 2:** Average Baseline Values of PROMIS Instruments and Respective Legacy Instruments

Domain	PROMIS (n)	Mean (SD) Median* [IQR]	Legacy Instrument (n)	Mean (SD) Median* [IQR]
**Pain Intensity**	Pain Intensity (*n* = 60)	53.7* [52.3–59.6]	Numerical Rating Scale (*n* = 44)	5.73 (1.97)
**Pain Interference**	Pain Interference (*n* = 68)	66.23 (6.06)	SF-36 Bodily Pain (*n* = 69)	31* [22–41]
			Brief Pain Inventory (*n* = 66)	5.95 (2.52)
**Pain Behavior**	Pain Behavior (*n* = 68)	60.82 (4.22)	Pain Catastrophizing Scale (*n* = 66)	1.58* [0.85–2.62]
			Fear Avoidance Belief Questionnaire (*n* = 68)	70.5* [57–80]
**Functional Status**	Physical Function (*n* = 68)	31.9* [27.2–36.5]	Roland-Morris Disability Questionnaire (RMDQ) (*n* = 63)	18* [13–21]
**Depression**	Depression (*n* = 68)	52.5 (10.77)	Patient Health Questionnaire (PHQ)-4 Depression (*n* = 63)	1* [0–3]
**Anxiety**	Anxiety (*n* = 68)	59.01 (9.99)	Patient Health Questionnaire (PHQ)-4 Anxiety (*n* = 63)	1* [0–3]
**Fatigue**	Fatigue (*n* = 64)	61.01 (958)	Functional Assessment of Chronic Illness Therapy (FACIT) Fatigue Subscale (*n* = 64)	25.81 (10.12)
**Sleep**	Sleep Disturbance (*n* = 61)	57.80 (13.03)	Medical Outcomes Study (MOS) Sleep Scale (*n* = 70)	45.66 (19.70)
**Social**	Social Isolation (*n* = 63)	46.3* [36.5–56.9]	MOS Social Support Survey (*n* = 63)	4.61* [3.53–5]

* = Median

The table includes only the completed instruments. Incomplete instruments, as described in the text, were not included in analyses. There was no non-response since the research team was administering all instruments

Based on previous research, domains from PROMIS instruments that represent the impact of back pain in older adults were selected to be administered as CAT in this study [10, 18]. Most of the CAT-administered instruments ranged from four to 12 items. These PROMIS (and corresponding legacy) instruments are listed in Table 2 in the following domains: pain intensity, pain interference, pain behavior, functional status, depression, anxiety, fatigue and sleep. With exception of physical function that does not include a time frame and the social health banks that reference “lately”, all PROMIS instruments reference the ‘last 7 days’. All PROMIS instruments, except for pain behavior, use a rating scale with five response options that reflect intensity or frequency. Pain behavior uses six response options with the lowest response “had no pain”. All PROMIS instruments are designed to produce a score where higher values indicate a greater presence of the construct being measured. PROMIS instrument results are reported as T-scores, with a mean of 50 and a standard deviation of 10 normed on the 2000 U.S. census general population [19]. For example, a patient with a score of 72 on the PROMIS Anxiety instrument indicates the patient is reporting levels of anxiety that are more than two standard deviations above the general population.

The legacy instruments, by domain, that were used in this study are described below. Pain intensity (over the last 7 days) was assessed using the NRS-PI measure. NRS-PI is a unidimensional 11 point scale measure of pain intensity in adults where the end points are the extremes of no pain (0) and worst pain (10) [20]. Pain interference was assessed with two legacy measures, the Short Form Health Survey (SF-36, Bodily Pain) and BPI. The SF-36 is a 36-item patient-reported survey to measure health status, and consists of eight scaled scores, which are the weighted sums of the questions in each section. A lower score indicates more disability [21]. The BPI is a measure of pain related functional impairment, and measures pain interference with seven daily activities, including general activity, walking, work, mood, enjoyment of life, relations with others, and sleep. It is scored as the mean of the seven interference items, that can be used if > 50% of the total items have been completed [22]. Pain behavior was assessed with two legacy measures, the Pain Catastrophizing Scale (PCS) and Fear Avoidance Belief Questionnaire (FABQ)*.* The PCS is a self-reported measure, where patients answer questions about how they feel and what they think about when they are in pain. The measure consists of 13 items scored from 0 to 4, resulting in a total possible score of 52. The higher the score, the more catastrophizing thoughts are present [23]. The FABQ measures patients’ fear of pain and consequent avoidance of physical activity because of their fear, and it consists of 16 items in which a patient rates their agreement with each statement on a 7-point Likert scale (0 = completely disagree, 6 = completely agree) with a maximum score of 96. The higher the score, the more strongly fear avoidance beliefs are held [24]. Functional status was assessed using the Roland-Morris Disability Questionnaire (RMDQ). The RMDQ is a measure of disability where patients are asked to read a list of 24 sentences and to place a tick against appropriate questions based on how they feel each sentence describes them today. The RMDQ is scored by adding up the number of items the patient has ticked, with a maximum score of 24, where higher scores reflect greater levels of disability [25]. Depression and anxiety symptom severity was assessed using the ‘Patient Health Questionnaire-4’ (PHQ-4) for depression and anxiety respectively. The PHQ-4 is a 4-item inventory (two questions on anxiety and two questions on depression) rated on a 4-point Likert-type scale ranging from ‘not at all’ with a score of zero to ‘nearly every day’ with a score of three. The total score is the sum of the four items with higher scores indicating more severe anxiety and depression symptoms [26]. Fatigue was assessed using the Functional Assessment of Chronic Illness Therapy (FACIT) Fatigue Subscale which is a short 13-item, measure of an individual’s level of fatigue during their usual daily activities over the past week. Subject responses are on a four-point Likert scale (0 = not at all and 4 = very much). Final scores are the sum of responses to the items and range from 0 to 52, where higher scores represent less fatigue [27]. Sleep was assessed with the Medical Outcomes Study (MOS) Sleep Scale which is a 12 item self-report sleep measure that contains 7 subscales and 2 overall index scores (a 6-item and a 9-item index) to assess important dimensions of sleep including initiation, maintenance, respiratory problems, quantity, perceived adequacy and somnolence. Higher scores indicate more of the concept being measured [28]. Social support was assessed with the MOS Social Support Survey (MOS SSS) which is a 19-item self-administered survey that covers four subscales (emotional/informational support, tangible support, positive social interaction, and affection), and an overall support index score is calculated and transformed to a 0–100 scale. A higher score for an individual scale or for the overall support index indicates more support is available [29]. It should be noted that unlike measures of social support (MOS SSS) that generally seek information about an individual's perception of the availability of support, the PROMIS Social Isolation instrument assesses perceptions of being avoided by or disconnected from others.

### Demographic and clinical variables

In addition to the instruments outlined above, the following data were obtained from the electronic medical record: age; sex; race (White, Black, other, declined); ethnicity (Hispanic vs non-Hispanic); pain duration; body mass index (BMI) [30]; current (at the time of recruitment) mental health conditions (depression, anxiety and/or PTSD) that are actively being treated (by medications or therapy**)**, medications prescribed at the time of recruitment (antidepressants, anxiolytics and/or analgesics including acetaminophen, NSAIDs and opioids); the Charlson comorbidity index [31]; percent service connection due to a musculoskeletal condition (this is a calculated disability rating given to a Veteran based on the severity of their service-connected conditions, and which determines their disability compensation and eligibility for VA benefits); and history of prior epidural steroid injection at least 3 months prior to enrollment.

### Statistical analysis

Descriptive statistics included means and standard deviations (SD) or median and interquartile range [IQR] for continuous variables depending on normality of the data, and n (percentages) for categorical variables [32]. Assumptions for parametric tests were not met; therefore, non-parametric statistical analyses used Spearman’s Rank-Order Correlations (rho) to measure the strength of association between ranked scores for PROMIS and legacy instruments and also provided an estimate of convergent and discriminant validity. Convergent validity refers to the degree to which two measures of constructs that theoretically should be related, are in fact related; it is estimated using correlation coefficients (rho) [33]. Discriminant validity refers to the degree to which two measures that should not theoretically be related indeed are not highly correlated [34]. The strength of correlation was defined as follows: weak (rho = 0.20–0.39); moderate (rho = 0.40–0.59); strong (rho = 0.60–0.79); very strong (rho = 0.80–1.0) [35]. We considered an additional category for very weak (rho = 0.00–0.19). When interpreting the strength of the relationship, we evaluate the absolute value of rho. Using these ranges for strength of association, we expect that the convergent validity would be strong or very strong; whereas the discriminant validity would be very weak, weak, or moderate. Discriminant validity should be less than the convergent validity.

Mann-Whitney U tests compared the administration length (time to completion) between PROMIS and legacy instruments, by domain. Only completed instruments or domains were included in the analyses. *P* < .05 was considered to indicate statistical significance. All analyses were conducted using Stata 14 [36].

## Results

We recruited 71 participants with cLBP with or without leg pain who met our inclusion criteria who agreed to participate in this study. The median age [IQR] of the sample was 67 (63–71) years and 67 (94.4%) were men. Fifty-four (76%) were Caucasian and 12 (17%) were African American; 68 (95.8%) were non-Hispanic. On average, participants were obese with a median BMI [IQR] of 30.98 (27.07–35.04). Forty-two (59.2%) participants had one or more documented mental health condition based on chart review (anxiety, depression and/or PTSD) and 45 (63.4%) participants were receiving a psychotropic medication (antidepressant or anxiolytic) at the time of enrollment. Regarding pain characteristics, 25 (35.2%) participants reported isolated back pain without any peripheral joint involvement, whereas the remainder of participants had multi-site pain including lumbar spine. Forty-five (63.4%) participants had back pain duration of at least 5 years with 93% reporting associated leg pain. Sixty-seven (94.4%) of the participants were using analgesic medications including acetaminophen, non-steroidal anti-inflammatory drugs and/or opioids. Additional demographics are summarized in Table 1. The most common ICD-9/10 diagnoses that were associated with the ESI procedure included: degenerative arthritis, degenerative disc disease, spinal stenosis and lumbago.

### Baseline PROMIS and legacy measures

While the number of participants recruited for this study was 71, the number of completed instruments (n) varied by instrument and are listed in Table 2. For PROMIS instruments, the mean number of administered items per domain ranged from four to six except for pain intensity where three items were administered in all cases. Three domains assessed pain: intensity, interference, and behavior. Pain behavior, as assessed using PROMIS, had a mean (SD) of 60.82 (4.22). This domain was correlated with two legacy instruments: PCS and FABQ with medians [IQR] of 1.58 [0.85–2.62] and 70.5 [57–80], respectively. Functional status was measured using PROMIS Physical Function with median [IQR] of 31.9 [27.2–36.5] and its corresponding legacy measure was the RMDQ with a median [IQR] score of 18 [13–21]. The baseline values of the remaining domains (depression, anxiety, fatigue, sleep disturbance and social) for PROMIS and their corresponding legacy instruments are listed in Table 2.

### Convergent validity

Table 3 provides the Spearman’s rank-order correlations between all PROMIS and legacy instrument scores. The validity diagonal (as highlighted in Table 3) contains the highest correlation coefficients across the row and column for a particular measured domain (PROMIS instruments across rows and legacy instruments in columns) with exception of PROMIS Pain Intensity, PROMIS Pain Behavior, PROMIS Physical Function, PROMIS Depression and PROMIS Social Isolation where the highest correlation was with a legacy measure different than their corresponding domain instrument. We found moderate convergent validity (rho = 0.51–0.59) in the domains for PROMIS Pain Intensity, PROMIS Pain Interference, and PROMIS Pain Behavior (PROMIS Pain Behavior in regards to PCS and not FABQ); strong convergent validity (rho = 0.61–0.76) for domains of PROMIS Physical Function and PROMIS Depression; very strong convergent validity for domains of PROMIS Anxiety, PROMIS Fatigue and PROMIS Sleep Disturbance (rho = 0.80–0.85). There was weak convergent validity (rho = 0.31–0.34) for the PROMIS Social Isolation and for the PROMIS Pain Behavior domain with respect to FABQ.

**Table 3:**
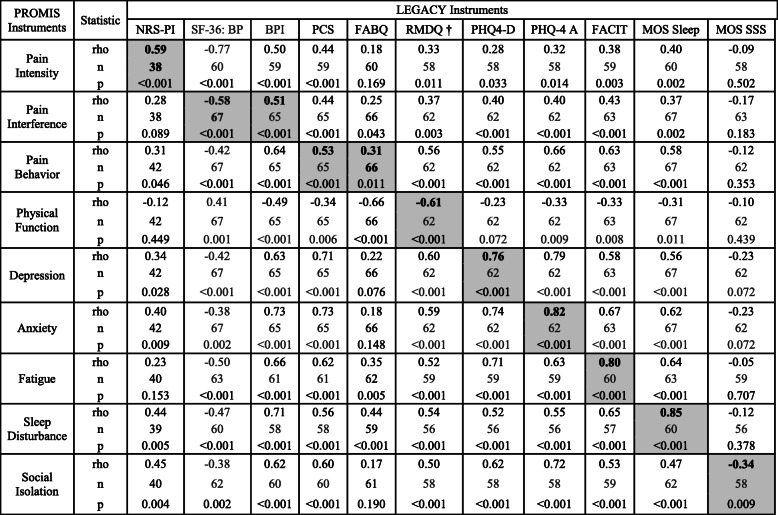
Spearman Rank Order Correlations Between PROMIS and Legacy Instruments

*NRS-PI* Numerical Rating Scale for Pain Intensity, *SF-36: BP* Medical Outcomes Study Short-Form 36: Bodily Pain Domain, *BPI* Brief Pain Inventory, *PCS* Pain Catastrophizing Scale, *FABQ* Fear Avoidance Beliefs Questionnaire, *RMDQ* Roland Morris Disability Questionnaire, *PHQ-4-D* Patient Health Questionnaire 4 Depression Subscale, *PHQ4-A* Patient Health Questionnaire 4 Anxiety Subscale, *FACIT* Functional Assessment of Chronic Illness Therapy – Fatigue, *MOS Sleep* Medical Outcomes Study – Sleep Scale, *MOS SSS* Medical Outcomes Study Social Support Survey; *rho* Spearman rank order correlation coefficient; *n* Number of pairs

Highlighted measures provide convergent validity. Strength of correlation: weak (*r* = 0.20–0.39); moderate (*r* = 0.40–0.59); strong (*r* = 0.60–0.79); very strong (*r* = 0.80–1.0) [35]

* = *p* < 0.05, ** = *p* < 0.01, † = Inverted scores

### Discriminant validity

Table 3 provides the discriminant validity which can be found in the off-diagonal cells. Using the categories for strength of association, we find that 63 of 88 discriminant validities in the off-diagonal range from very weak to moderate correlations. The exceptions that stand out are in the domains for Pain Intensity with SF-36: BP (rho = − 0.77), Pain Behavior with BPI, PHQ-4, and FACIT (rho = 0.63–0.66), Physical Function with FABQ (rho = − 0.66), Depression with BPI, PCS, RMDQ, and PHQ-4 A (rho = 0.60–0.79), Anxiety with BPI, PCS PHQ4-D, FACIT, and MOS Sleep (rho = 0.62–0.74), Fatigue with BPI, PCS, PHQ4-D, PHQ-4 A and MOS Sleep (rho = 0.62–0.71), Sleep Disturbance with BPI and FACIT (rho = 0.65–0.71), and Social Isolation with BPI, PCS, PHQ4-D and PHQ-4 A (rho = 0.60–0.72).

If we define positive evidence for convergent/discriminant validity as the definition that convergent validity has a higher correlation compared to all discriminant validities for that measure, four (44.4%) PROMIS instruments (Pain Interference, Anxiety, Fatigue and Sleep Disturbance) meet this criteria and three (33.3%) others (Pain Intensity, Physical Function, and Depression) miss this criteria by one comparison. Evaluating the Legacy instruments, seven (63.6%) instruments (NRS-PI, RMDQ, PHQ4-D, PHQ-4 A, FACIT, MOS Sleep and MOS SSS) meet this criteria and one (9.1%) additional instrument (SF-36) missed this criteria by one comparison.

### Time to complete

The median time to complete each CAT-administered PROMIS instrument ranged from 23 s to 58 s. The time to complete individual legacy instruments ranged from 13 s to 6 min and 7 s. Administration duration for the NRS-PI was unavailable since it was completed using a paper and pen modality and time to complete was not measured. The median total time participants needed to complete all the PROMIS instruments was shorter than that needed to complete legacy items across most domains, with total median time of 8 min 50 s vs 29 min 14 s respectively, *p* < 0.001 (Table 4). The median time to complete PROMIS Depression and Anxiety screen was longer than legacy Patient Health Questionnaire (PHQ)-4 Depression and Anxiety (35 vs 13 s; *p* < 0.001 for depression and 39 vs 15 s; *p* < 0.001 for anxiety, respectively).

**Table 4:** Median Time to Complete PROMIS vs Legacy Instruments (minutes:seconds)*

DOMAIN	PROMIS INSTRUMENT	PROMIS min:sec	LEGACY INSTRUMENT	LEGACY min:sec
**Pain Intensity**	Pain Intensity	0:23	NRS-PI	N/A
**Pain Interference**	Pain Interference	0:58	SF-36:BP	5:20
			BPI	3:20
**Pain Behavior**	Pain Behavior	0:38	PCS	2:17
			FABQ	6:07
**Functional Status**	Physical Function	0:41	RMDQ	2:13
**Depression**	Depression	0:35	PHQ4: Depression	0:13
**Anxiety**	Anxiety	0:39	PHQ4: Anxiety	0:15
**Fatigue**	Fatigue	0:32	FACIT	1:24
**Sleep**	Sleep Disturbance	0:38	MOS Sleep	3:06
**Social**	Social Isolation	0:38	MOS SSS	2:00
**Complete Battery**^a^	PROMIS Battery	8:50	LEGACY Battery^b^	29:14

*NRS-PI* Numerical Rating Scale for Pain Intensity, *SF-36* Medical Outcomes Study Short Form 36-Item Survey, *BPI* Brief Pain Inventory, *PCS* Pain Catastrophizing Scale, *FABQ* Fear Avoidance Belief Questionnaire, *RMDQ* Roland Morris Disability Questionnaire, *PHQ* Patient Health Questionnaire, *FACIT* Functional Assessment of Chronic Illness Therapy – Fatigue, *MOS Sleep* Medical Outcomes Study – Sleep Scale, *MOS SSS* Medical Outcomes Study Social Support Survey.

* All comparisons were significant with a *p* < 0.001

^a^ Median value for total administration length of PROMIS instruments and for total administration length of LEGACY measures across entire sample. Does not indicate a sum of individual instrument median values presented in table.

^b^ Legacy battery consisted of a greater number of individual measures than PROMIS, contributing to a longer time to completion as compared to PROMIS

The research staff documented in field notes that PROMIS instruments were easier for participants to complete due to consistent formatting and wording of the questions and the uniformity of the Likert-type scale responses available.

## Discussion

In this cross-sectional analysis we evaluated construct validity (with both convergent and discriminant) and time to completion of PROMIS and legacy instruments in corresponding domains that are relevant to older adults with cLBP with and without leg pain. The PROMIS instrument scores were correlated with legacy instrument scores of similar domains, per the hypothesis, with moderate to very strong convergent validity in all domains except for a weak convergent validity for the social functioning domain. The two domains for PROMIS instruments that stood out with poor construct validity (a comparison between convergent and discriminant as described in the analysis section) were Pain Behavior and Social Isolation; for legacy instruments, we found poor construct validity for BPI, PCS, and FABQ. PROMIS instruments were more efficient in evaluating nine different domains as compared to most of the legacy measures. To our knowledge, this is the first study that validates the psychometric properties and feasibility of applying these PROMIS and legacy instruments in an older Veteran population with cLBP.

Our results are consistent with literature focused on patients with chronic back pain, where PROMIS instruments correlated well with legacy measures [37–40]. In a retrospective review of an outcomes database, PROMIS Pain Interference, Physical Function, and Pain Intensity instruments correlated strongly with traditional disability measures in patients with back and neck pain [41]. In the present sample of older Veterans, results were similar to those previously reported in older Veterans who are known to have additional burden of physical and psychiatric comorbidity [42, 43]. Mental health conditions can impact patients’ perceptions of their musculoskeletal disease and influence their self-reported outcome measures. A recent study showed that patients with symptomatic glenohumeral arthritis with worse PROMIS Depression and Anxiety scores as compared to those with scores in the normal range, had lower functional outcome and higher pain scores [44]. In this study, the severity of mental health comorbidity was evaluated using PROMIS Depression and Anxiety instruments which correlated strongly with legacy PHQ-4. Consistent with results reported by Kohan et al., our sample showed that PROMIS Depression and Anxiety domains had strong correlation to BPI and a moderate inverse correlation with RMDQ, reflecting a higher relationship with pain scores and lower, inverse relationship to functional status.

The correlation between PROMIS and legacy social constructs was weak, and one potential reason for this is that social dimensions of health are multifactorial and multidimensional, whereby PROMIS instruments may not have mapped directly to the legacy measures we selected. The items and description of the PROMIS Social Isolation Instruments assess feelings (e.g., “I feel isolated from others.”), aside from two that ask: “I find that friends or relatives have difficulty talking with me about my health” and “People get the wrong idea about my situation.” Whereas, the MOS Social Support Survey asks whether the person has individuals they can ask for emotional and physical support (but does not indicate that they actually use said social support). An individual can indicate they have social support (e.g., high MOS scores, indicating that they have friends and family they could ask for support), but do not feel it (e.g., high PROMIS Social Isolation, indicating that they feel like the same friends and family avoid them); therefore, the lack of significant correlation may not be surprising.

In busy clinical and research settings, it is important to identify valid and efficient tools to collect PROs. Some health care settings accomplish this prior to the visit (via email or on the internet) or this information may be captured while patients are waiting for their appointment on the day of the visit [12, 13, 45, 46]. Incorporating PRO, legacy or PROMIS, is increasingly becoming standard of practice [47]. Deciding which instrument to use and ability to compare outcomes with each other can be guided by using PROsetta stone (http://www.prosettastone.org/). This resource links ‘legacy’ instrument results to the PROMIS metric. In general, our results suggest that PROMIS instruments are a practical choice to measure multiple PROs prior to or during a clinical visit. Because of the CAT administration, as well as the lower respondent burden (due to fewer items needed to be completed: 4–6 items required for precise measurement of health-related constructs using CAT) (http://www.healthmeasures.net/explore-measurement-systems/promis/intro-to-promis), PROMIS is a promising choice. Looking at individual domains, PROMIS instruments were completed faster than legacy measures in all domains except for depression and anxiety which were faster using legacy measures – the 4-item questionnaire PHQ-4 (Table 4). This is likely due to the short and brief structure of the PHQ-4 questionnaire that was selected in our study. PROMIS depression and anxiety domains could have been completed faster than legacy measures if PHQ-9 or another more comprehensive assessment of depression or anxiety was selected for comparison. Our results suggest that busy clinics and researchers (who don’t want to overwhelm their study participants with lengthy, burdensome assessments) might consider using PROMIS to assess domains appropriate for their population of interest. Nonetheless, future research is needed to evaluate whether and how collection of PROs actually modifies outcomes for patients and if/how they change the flow of practice and decision making for clinicians.

Our study has several strengths. We were able to recruit a population of older Veterans with multiple chronic conditions—including psychological comorbidities—and complete both sets of instruments in multiple (nine) PRO domains. It must be noted that not all instruments were completed due to interruption when called for the procedure, as might be expected in a busy clinical setting. Part of the success of completing so many instruments was due to research staff administering the surveys on the computer with the participant (Veterans did not complete the surveys independently). Future research should evaluate different modalities of PRO delivery to see if older Veterans can successfully complete the instruments independently, and which modes of assessment (or delivery) are preferred. Recent literature suggests that older adults do not have difficulty completing self-reported instruments using varying platforms [48, 49]. Moreover, PROMIS instruments appear to function well in older adults with cognitive impairment. For instance, in a study conducted on community dwelling older adults with varying degrees of cognitive impairment, Levi et al. evaluated the utility of PROMIS Depression Scale compared to legacy depression instruments (Montgomery-Asberg Depression Rating Scale, Geriatric Depression Scale (GDS), and GDS-Short Form) and found no statistically significant differences in depression scores by cognitive status group between the instruments [43].

Limitation of our study included a relatively small sample size, at a single site at the Dallas VA PM&R Spine clinic. Results of our study cannot be generalized to a non-Veteran population. Veterans tend to have more functional disability than the general population [42]. We did not have granular data on specific medications used (these were grouped broadly into categories of analgesic and psychotropic medications). While the research team attempted to work closely with the nursing and PM&R team, the shear duration to complete both PROMIS and legacy in all these domains was, at times, disruptive to clinical flow. Duration to complete certain measures may have been affected by interruptions by nursing staff taking vital signs or research staff clarifying or assisting with questions. The total time to completion for the legacy battery of instruments was longer, in part, because we included several instruments for each domain and these were not CAT. Additional research is warranted to evaluate administration and implementation of PRO in busy clinics by non-research staff [12].

Another potential limitation is the selection of legacy and corresponding PROMIS instruments. While we used the literature and our clinical experience to guide this selection, for example, FABQ legacy instrument may not have been the most appropriate to correlate with the Pain Behavior PROMIS instrument (see Table 3). However, we learned from evaluating discriminant validity that FABQ correlates more strongly with the Physical Function PROMIS instrument which can be helpful in clinical and research settings. This finding also highlights that pain catastrophizing and fear avoidance are fundamentally different behavioral constructs and may be better captured with different PROMIS measures (Pain Behavior and Physical Function, respectively).

## Conclusion

In conclusion, we found that PROMIS instruments, especially for pain, depression, anxiety, fatigue and sleep domains, have strong convergent validity in older Veterans with chronic back pain with and without leg pain. Given time efficiency of using PROMIS, along with strong construct validity (77.7% for PROMIS vs 72.7% for legacy) in this study, PROMIS instruments are a practical choice for measuring multidimensional PROs for both research and clinical purposes.

## Data Availability

We are ready to share these data with colleagues after appropriate institutional, ethics and patient privacy requirements have been met. Please contact the corresponding author for data and material requests: Una E. Makris, MD, MSc (una.makris@utsouthwestern.edu; una.makris2@va.gov).

## Abbreviations

cLBP: Chronic Low Back Pain
PROMIS: Patient Reported Outcomes Measurement Information System
TTC: Time to Complete
PRO: Patient Reported Outcomes
IQR: Interquartile Range
NIH: National Institutes of Health
CAT: Computer Adaptive Testing
IRT: Item Response Theory
PM&R: Physical Medicine & Rehabilitation
ESI: Epidural Steroid Injection
BPI: Brief Pain Inventory
NRS-PI: Numerical Rating Scale for Pain Intensity
SF-36: Medical Outcomes Study Short Form 36-Item Survey
PCS: Pain Catastrophizing Scale
FABQ: Fear Avoidance Belief Questionnaire
RMDQ: Roland Morris Disability Questionnaire
PHQ: Patient Health Questionnaire
FACIT: Functional Assessment of Chronic Illness Therapy – Fatigue
MOS Sleep: Medical Outcomes Study – Sleep Scale
MOS SSS: Medical Outcomes Study Social Support Survey
PTSD: Post-traumatic stress disorder
BMI: Body Mass Index
NSAIDs: Non-steroidal Anti-inflammatory drugs
SD: Standard Deviation
ICD: International Classification of Disease
